# Supplementary material for: The Fluorescent Quenching Mechanism of N and S Co-Doped Graphene Quantum Dots with Fe3+ and Hg2+ Ions and Their Application as a Novel Fluorescent Sensor
Source: Nanomaterials (Basel). 2019 May 13;9(5):738. doi: 10.3390/nano9050738 (PMC6566331; doi:10.3390/nano9050738)
Supplement: Supplementary file 1 [file nanomaterials-09-00738-s001.pdf]

Supplementary Information

The Fluorescent Quenching Mechanism of N and S Co-Doped Graphene Quantum Dots with Fe^{3+} and Hg^{2+} Ions and Their Application as a Novel Fluorescent Sensor

Yue Yang [1], Tong Zou [2], Zhezhe Wang [1], Xinxin Xing [1], Sijia Peng [2], Rongjun Zhao [1], Xu Zhang [2] and Yude Wang [2,3,*]

Table 1. The atomic populations of Fe^{3+}@N, S-GQDs and Hg^{2+}@N,S-GQDs.

Materials		Atomic Populations (Mulliken)					Bond Population		*d* (Å)
		s	***p***	***d***	**Total**	**Charge (e)**			
**Fe^{3+}**	**C4**	1.20	2.98		4.18	-0.18	C1-O	0.48	1.43
	**C9**	1.09	3.02		4.12	-0.12	C1-S	0.53	1.76
	**C10**	1.26	2.92		4.18	-0.18	C2-S	0.64	1.70
	**N**	1.40	3.84		5.23	-0.24	C2-N	0.88	1.39
	**O**	1.81	4.60		6.42	-0.42	C3-N	0.83	141
	**S**	1.63	3.60		5.22	0.78	C3-O	-0.05	2.63
	**Fe**	0.28	-0.10	7.16	7.34	0.66	Fe-O	-0.12	2.64
							Fe-S	0.17	2.033
							Fe-N	-0.17	2.80
**Hg^{2+}**	**C4**	1.36	2.83		4.19	-0.19	C1-O	0.32	1.57
	**C9**	1.07	3.07		4.14	-0.14	C1-S	0.43	1.76
	**C10**	1.03	2.75		3.77	0.23	C2-S	0.59	1.71
	**N**	1.39	3.87		5.25	-0.25	C2-N	0.86	1.39
	**O**	1.73	4.55		6.28	-0.28	C3-N	0.91	1.41
	**S**	1.70	3.50		5.20	0.80	C3-O	0.50	1.51
	**Hg**	1.87	0.01	10.0	11.87	0.13	Hg-O	-0.07	3.37
							Hg-S	-0.06	3.85
							Hg-N	-0.13	3.24

Table S2 Recovery of Fe^{3+} and Hg^{2+} detection in drinking water samples.

**Sample**	**Spiked concentration**	**Detected concentration**	**Recovery ± RSD (%)**
Fe^{3+} in Drinking water	700 nM	654 nM	93 ± 4.1
	1 μM	1.27 μM	127 ± 5.3
	3 μM	3.63 μM	121 ± 6.6
Hg^{2+} in Drinking water	50 nM	62 nM	124 ± 3.4
	100 nM	114 nM	114 ± 4.6
	300 nM	284 nM	95 ± 7.6

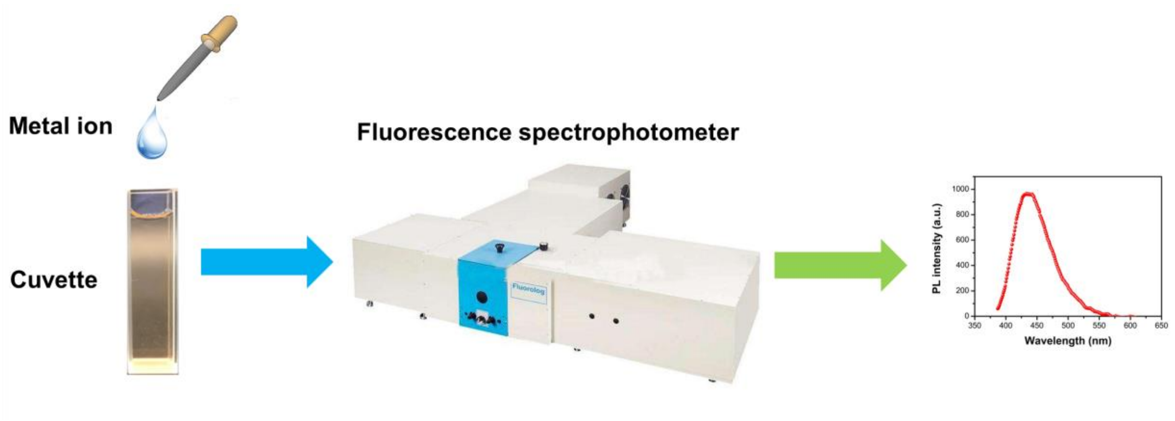

Figure S1. The schematic diagram of detection device geometry and testing process.

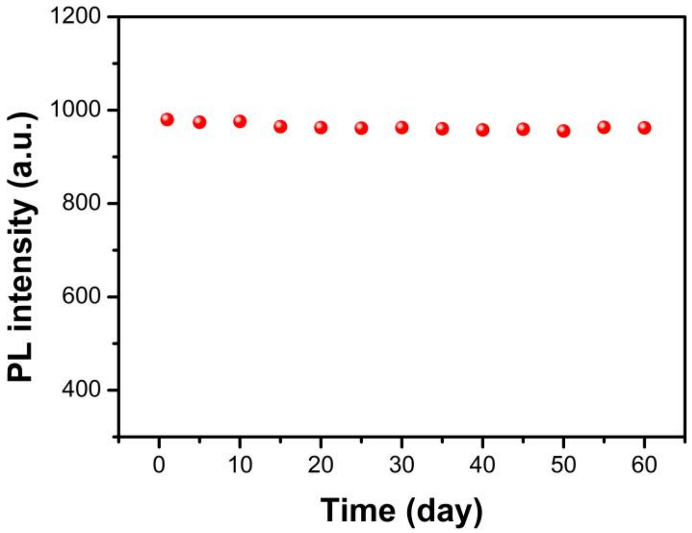

Figure S2. The stability of fluorescence intensity of as-synthesized N, S-GQDs solutions.

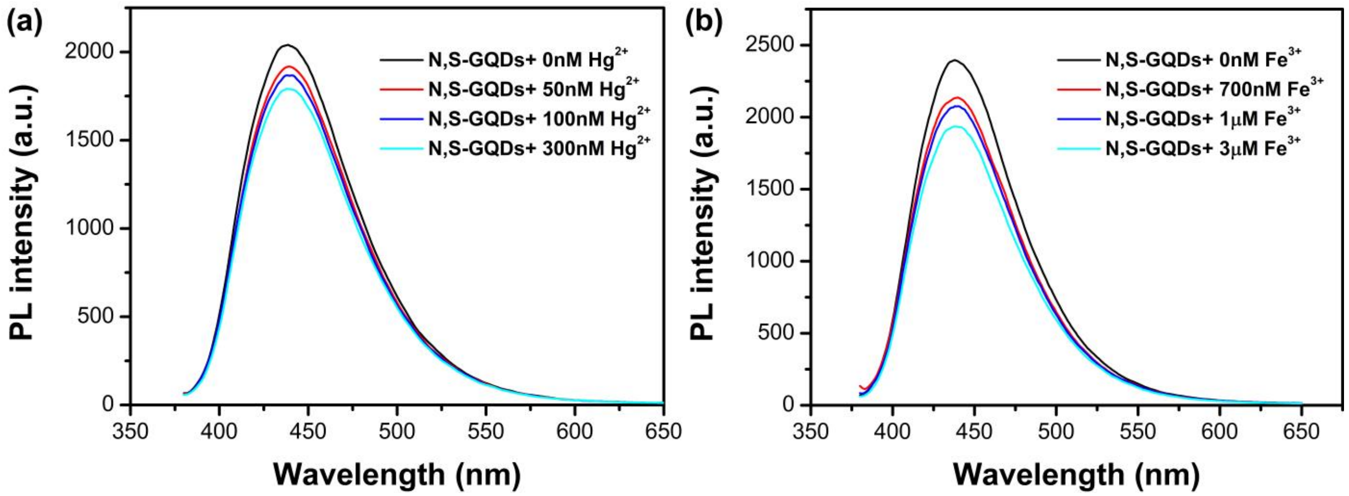

Figure S3. The fluorescence intensity of N, S-GQDs in real sample detection.
